# Impact of a non‐fluoridated microcrystalline hydroxyapatite dentifrice on enamel caries progression in highly caries‐susceptible orthodontic patients: A randomized, controlled 6‐month trial

**DOI:** 10.1111/jicd.12399

**Published:** 2019-01-30

**Authors:** Ulrich Schlagenhauf, Karl‐Heinz Kunzelmann, Christian Hannig, Theodor W. May, Helmut Hösl, Mario Gratza, Gabriele Viergutz, Marco Nazet, Sebastian Schamberger, Peter Proff

**Affiliations:** ^1^ Department of Conservative Dentistry and Periodontology University Hospital Wuerzburg Wuerzburg Germany; ^2^ Department of Conservative Dentistry and Periodontology Ludwig‐Maximilians‐University Munich Germany; ^3^ Clinic of Operative and Pediatric Dentistry Medical Faculty Carl Gustav Carus Technical University Dresden Dresden Germany; ^4^ Society for Biometry and Psychometry Bielefeld Germany; ^5^ Department of Orthodontics University Hospital Regensburg Regensburg Germany; ^6^ Department of Orthodontics University Hospital Wuerzburg Wuerzburg Germany; ^7^ Department of Orthodontics Ludwig‐Maximilians‐University Munich Germany; ^8^ Department of Orthodontics Johann‐Wolfgang‐Goethe‐University Frankfurt Germany

**Keywords:** dentifrice, enamel caries, fluoride, hydroxyapatite, orthodontic therapy

## Abstract

**Aim:**

The aim of the present randomized, controlled trial was to compare the impact of the regular use of a fluoride‐free microcrystalline hydroxyapatite (HAP) dentifrice and a 1400 ppm fluoride control dentifrice on caries progression in 150 highly caries‐active orthodontic patients.

**Methods:**

The primary outcome was the occurrence of lesions with International Caries Detection and Assessment System (ICDAS) ≥code 1 on the vestibular surfaces of teeth 15‐25 within 168 days after fixation of orthodontic brackets. Secondary outcomes were lesion development ICDAS ≥code 2, the plaque index, and the gingival index.

**Results:**

In total, 147 patients were included in the intent‐to‐treat (ITT) analysis; 133 finished the study per protocol (PP). An increase in enamel caries ICDAS ≥code 1 was observed in 56.8% (ITT) and 54.7% (PP) of the HAP group participants compared with 60.9% (ITT) and 61.6% (PP) of the fluoride control group. Non‐inferiority testing (ITT and PP) demonstrated the absence of a significant difference between the groups. No significant differences in secondary outcomes were observed between the groups.

**Conclusion:**

In highly caries‐active patients, the impact of the regular use of a microcrystalline HAP dentifrice on caries progression is not significantly different from the use of a 1400 ppm fluoride toothpaste (ClinicalTrials.gov: NCT02705456).

## 1 INTRODUCTION

Recent findings, mostly derived from in vitro studies, have suggested that microcrystalline hydroxyapatite (HAP) particles might be suitable candidates for the prevention of demineralization and the stimulation of remineralization processes on enamel and dentine surfaces.^1^, ^2^, ^3^ Furthermore, in an in situ study, the use of (a pure HAP was used) HAP microcluster‐containing mouthrinse significantly reduced bacterial colonization on bovine enamel slabs worn intraorally by healthy volunteers.^4^

Hannig and Hannig put these in situ and in vitro findings into a more comprehensive perspective by stating that established physiological tooth wear constantly releases HAP particles into the oral environment, which might subsequently interfere with demineralization and remineralization processes, as well as with the metabolism of the oral microbiota at the tooth‐bacterial biofilm interface.^5^ The impact of microcrystalline HAP as an ingredient in dentifrices has been positively evaluated in controlled clinical trials on dentinal hypersensitivity,^6^, ^7^, ^8^, ^9^ and parameters of periodontal health.^10^ To date, however, comparable data regarding the caries‐inhibiting properties of HAP toothpastes are lacking. As orthodontic therapy with fixed appliances is known to be associated with an increased incidence of the overgrowth of a caries‐promoting microbiota^11^ and the development of white spot enamel caries lesions,^12^, ^13^, ^14^ the aim of the of the present study was to assess the caries‐inhibiting impact of the regular use of a fluoride‐free HAP dentifrice in this particular group of patients with caries risk. Due to the abundant evidence for the caries preventive efficacy of fluorides,^15^, ^16^ clinical caries studies might no longer involve a true negative control for obvious ethical reasons. Thus, a non‐inferiority trial was conducted. The study hypothesis to be tested was whether or not the regular use of the HAP test dentifrice was inferior to the regular use of a fluoridated control in terms of caries prevention.

## 2 MATERIALS AND METHODS

The investigation was designed as a multicenter, prospective, parallel‐group, two‐arm, double‐blinded, randomized, clinical non‐inferiority trial to be performed at the German study centres Wuerzburg (leading study center), Regensburg, Munich, Dresden, and Frankfurt. The study protocol was prepared in accordance with the Declaration of Helsinki and met the good clinical practice criteria. It was approved by the ethics committee of the University of Wuerzburg (file no. 184/13) and was registered at ClinicialTrials.gov (identifier no.: NCT02705456).

### 2.1 Study design

**Figure 1:**
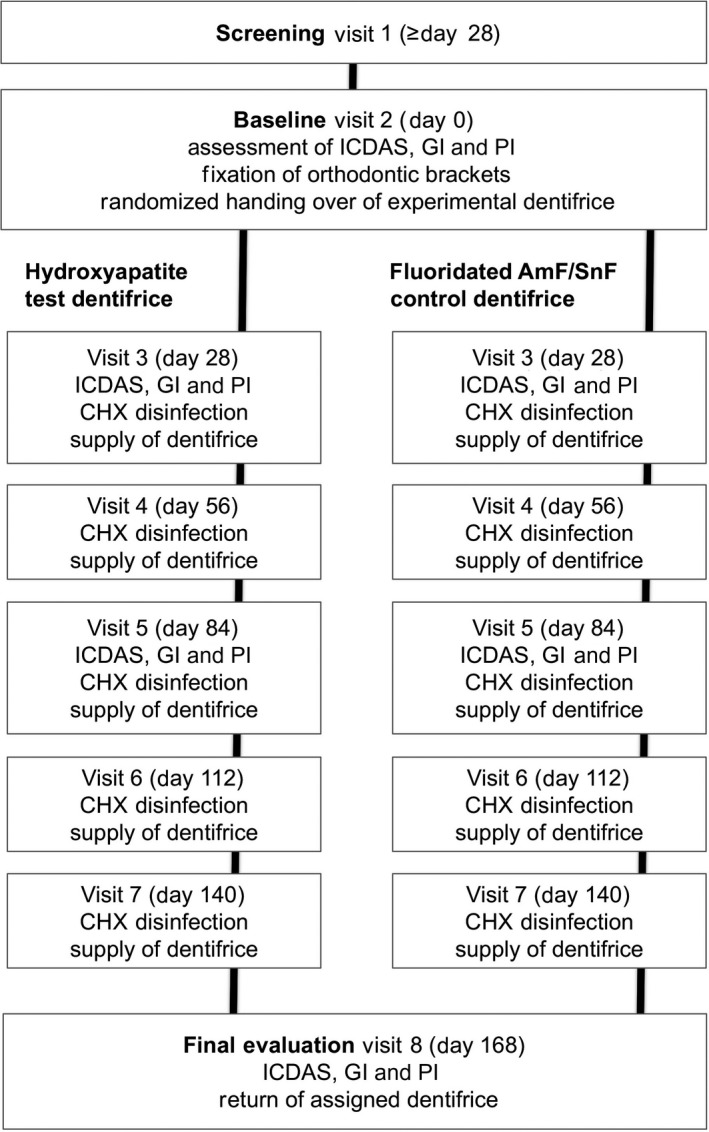
Schematic study design. CHX, chlorhexidine; GI, gingival index; ICDAS, International Caries Detection and Assessment System; PI, plaque index

The design of the study is schematically depicted in Figure 1. At visit 1 (−4 to −28 days prior to baseline), patients scheduled for orthodontic therapy were screened for study eligibility. Those meeting the eligibility criteria were asked to participate, and after providing informed consent, they were scheduled for the baseline visit 2 (day 0).At visit 2, the plaque index (PI) and the gingival index (GI) scores were recorded from the vestibular surfaces of teeth 15‐25, followed by professional tooth cleaning and the subsequent assessment of the vestibular enamel surfaces of teeth 15‐25 according to International Caries Detection and Assessment System (ICDAS) II criteria. Orthodontic brackets were then adhesively mounted to the vestibular surfaces. No sealants, fluoride varnishes, or any other caries‐preventive layers surrounding the brackets were applied. Using a randomization list, a supply of either the test dentifrice or the control dentifrice, calculated to be adequate for 4 weeks of 2× daily repeated toothbrushing, as well as a standardized electric toothbrush (Oral‐B Pulsar 35; Procter & Gamble GmbH, Schwalbach, Germany) to be used for the duration of the study, were given to the study patients. Practical training was provided for the dosing of the assigned dentifrice (2× daily a streak of approximately 1 g) and the use of the electric toothbrush, and the patients were instructed to return all the assigned toothpaste tubes at the next scheduled visit. At day 28, the sequence of recording the PI, GI, and ICDAS II scores was repeated, as described for visit 2. As an additional caries‐preventive measure, teeth 15‐25 were disinfected with a topically‐applied 1% chlorhexidine gel. Toothpaste tubes supplied at visit 2 were collected, and a new supply was provided for the next 4 weeks. At day 56 (visit 4), oral hygiene reinstruction was provided, as well as cleaning/disinfection procedures and return/handing over of the toothpaste supply, as described earlier. At day 84 (visit 5), the recording of the PI, GI, and ICDAS II scores and cleaning and disinfection were repeated, as described earlier. In addition to a new supply of toothpaste, a new electric toothbrush was also provided. At day 112 (visit 6) and day 140 (visit 7), the performed procedures were identical to those at day 56 (visit 4). At day 168 (visit 8), the final assessment of the PI, GI, and ICDAS II scores and the return of the study dentifrices were conducted, as described before. Furthermore, at each study visit, the patients were asked about the occurrence of important problems or unintended effects related or unrelated to the use of the study dentifrices.

### 2.2 Study population

The trial was performed in healthy adolescents and young adults who were scheduled for orthodontic therapy with fixed appliances.

#### 2.2.1 Inclusion criteria

The inclusion criteria were: (a) patients aged 11‐25 years; (b) scheduled orthodontic therapy with fixed appliances of at least 6 months’ duration; (c) placement of orthodontic brackets on the vestibular surfaces of teeth 15‐25; (d) regular (2× daily) oral home care with a toothbrush and toothpaste; and (e) caries‐promoting salivary counts of *mutans streptococci* ≥10^5^ c.f.u./mL, determined using the CRT bacteria test (Ivoclar Vivadent, Schaan, Liechtenstein).^17^

#### 2.2.2 Exclusion criteria

The inclusion criteria were: (a) untreated caries lesions of ICDAS code 3‐6 on any tooth; (b) treated carious lesions of ICDAS code 3‐6 on the vestibular surfaces of teeth 15‐25; (c) diseases or conditions or the regular use of related medications that interfere with salivary flow; (d) antibiotic therapy within the past 6 weeks before study participation or the necessity for antibiotic prophylaxis during dental interventions; and (e) known allergies to ingredients in the experimental dentifrices.

### 2.3 Interventions: Experimental dentifrices

#### 2.3.1 Test dentifrice

The test dentifrice (Karex Zahnpasta; Dr Kurt Wolff GmbH & Co. KG, Bielefeld, Germany) was provided by the sponsor of the study. It contained 10% microcrystalline HAP as the main caries‐preventive agent and the following ingredients: aqua, glycerol, hydrogenated starch hydrolysate, xylitol, hydrated silica, silica, aroma, cellulose gum, sodium methyl cocoyl taurate, *Helianthus annuus* seed oil, polyglyceryl‐3 palmitate, polyglyceryl‐6 caprylate, and *Usnea barbata* extract.

#### 2.3.2 Control dentifrice

A commercially‐available fluoridated toothpaste (meridol Zahnpasta; CP GABA GmbH, Hamburg, Germany) was used as a positive control. It contained amine fluoride and stannous fluoride at concentrations of 350 and 1050 ppm, respectively, as well as the following ingredients: aqua, sorbitol, hydrated silica, silica dimethyl silylate, hydroxyethylcellulose, polyethylene glycol (PEG)‐40, hydrogenated castor oil, cocamidopropyl betaine, aroma, sodium gluconate, PEG‐3 tallow aminopropylamine, saccharin, hydrochloric acid, potassium hydroxide, and CI 74160.

### 2.4 Primary outcome

The primary study outcome was the percentage of participants in each experimental group with a new occurrence of at least one enamel caries lesion ICDAS ≥code 1 on the vestibular surfaces of teeth 15‐25 during the observation period of 168 days.

### 2.5 Caries assessment

The occurrence of caries was evaluated visually on the vestibular surfaces of teeth 15‐25 according to ICDAS‐II criteria.^18^ The examination was performed at baseline, prior to the fixation of the orthodontic brackets, and was repeated after 28 days, 84 days,and 168 days. All teeth were professionally cleaned before each assessment. The development of a caries lesion ICDAS >code 3 during the course of the study on any tooth and observed at any visit was defined as an immediate study exit criterion.

### 2.6 Interexaminer reliability

To ensure interexaminer reliability, prior to the study onset all examiners were instructed to pass the ICDAS e‐learning course at the icdas.org website and were subsequently trained in person by an experienced expert (K.H.K.) to perform ICDAS assessments in reference patients. Grading skills were retrained three times during the course of the study using another Internet‐based ICDAS training tool. Interrater reliability analysis revealed a mean weighted ĸ = 0.75 for the first assessment run, which increased to ĸ = 0.80 for the final calibration, indicating “substantial agreement” among the different examiners throughout the study.^19^

### 2.7 Secondary outcomes

Secondary outcomes were the new occurrence of at least one enamel caries lesion ICDAS ≥code 2 on the vestibular surfaces of teeth 15‐25 and plaque coverage and gingival inflammation assessed by recording the PI and GI at baseline and at day 168.^20^, ^21^

### 2.8 Statistical analysis

The primary outcome measure was analyzed primarily for the per protocol (PP) population and repeated for sensitivity reasons for the intent‐to‐treat (ITT) population. The exact confidence limits (Clopper‐Pearson) were computed to test non‐inferiority.^22^ For the primary outcome measure, non‐inferiority was claimed if the upper limit of the one‐sided 95% confidence for the corresponding difference between test and control dentifrice was less than Δ (difference) ≤20%.

In addition, two‐sided Wilcoxon‐Mann‐Whitney tests were used for between‐group comparisons, and Friedman tests for within‐group comparisons, for secondary outcomes.

SAS 9.3 software package (SAS Institute, Cary, NC, USA) was used for the statistical evaluations.

### 2.9 Sample size calculation

Based on a reported caries incidence rate of approximately 60% in a preceding caries trial assessing orthodontic patients with fixed braces who were not being preselected for particular caries‐promoting risk factors,^13^ the likelihood for the occurrence of an ICDAS code 2 lesion during the 168‐day observation period in this cohort of caries‐risk individuals with elevated salivary numbers of caries‐promoting *mutans streptococci* was extrapolated to be *P *=* *80% for the control group using the fluoridated toothpaste. The difference between both experimental groups was not regarded to be clinically relevant and was set to Δ ≤ 20%. A sample size of 2 × 74 study patients was calculated to be sufficient to reject the null hypothesis, that the test dentifrice is inferior to the control dentifrice, using a non‐inferiority margin of Δ = 20% for the primary outcome measure and one‐sided, exact Fisher's test (α = 5%, power = 80%).

### 2.10 Blinded change of the primary outcome

A blinded analysis of the ICDAS data at the end of the study revealed that the overall observed occurrence of ICDAS lesions ≥code 2 in the study population was 29.3%, and therefore considerably lower than the anticipated value (*P *=* *80%) used for the sample size calculation. As the difference between the groups was not regarded to be clinically relevant and had been set in the study protocol to Δ ≤ 20%, a clinically meaningful verification of non‐inferiority was no longer warranted. Thus, the primary endpoint was changed to the more frequent overall occurrence of ICDAS lesions ≥code 1 (59.2%). We decided to keep the original primary endpoint as an additional secondary outcome in the statistical data analysis.

While it might have been debatable to keep the original non‐inferiority margin of Δ = 20% when switching the primary outcome of the trial, despite an overall incidence of the revised primary outcome (ICDAS lesion code 1) of only 60%, the subsequent analysis of the unblinded PP dataset revealed that the actual difference between both experimental groups was 6.2% in favor of the HAP test dentifrice with an exact upper one‐sided 95% confidence limit of 8.3% (i.e substantially lower than the preset non‐inferiority margin of Δ = 20%).

### 2.11 Blinding and randomization

The trial was designed to blind study patients and examiners to the group assignment. Both study dentifrices (test/control) were filled into neutral plastic tubes of identical shape and color by an independent, good manufacturing practice‐certified laboratory for cosmetics. Using block randomization with a block size of 4, a random list was generated to code label test and control tubes with consecutive unique identification numbers. The randomization of dentifrice assignment was stratified by the study center. Distribution of the experimental dentifrices to the study patients followed the sequence of the identification numbers and was performed by trained study nurses not involved in the examination of the study participants. To maintain blinding of examiners and study patients, the study patients were instructed not to discuss toothpaste‐related issues with the examiners, but with the study nurses only, who were also responsible for instructing the patients in efficacious oral hygiene and taking back the empty or unused dentifrice tubes at the subsequent visits. The number of study nurses varied between a minimum of one and a maximum of four per study center.

### 2.12 Interexaminer reliability

Grading skills were retrained three times during the course of the study using an Internet‐based ICDAS training tool. It confronted the examiners with a random sample of 40 pictures of upper premolars, canines, and incisors with surface integrity ICDAS codes 0‐3. In total, 50% of the pictures of a given sample were randomly presented in duplicates to evaluate the ability of the examiners to reproduce their own assessments.

Although up to three examiners were trained and calibrated at each study center before the onset of the trial, at four centres the bulk of the practical evaluations was performed by a single principal examiner (Munich: 100% of all visits, Frankfurt: 100%, Regensburg: 96%, and Wuerzburg: 96%) At the center in Dresden, the principal examiner performed 58% of all examinations, and the second examiner 38%. Although up to three examiners were trained and calibrated at each study center before the onset of the trial, at four centres, the bulk of the practical evaluations was performed by a single principal examiner (Munich: 100% of all visits, Frankfurt: 100%, Regensburg: 96%, Wuerzburg: 96%). At the center in Dresden, the principal examiner performed 58% of all examinations, and the second examiner 38%.

### 2.13 Number and severity of International Caries Detection and Assessment System score increases

The number and severity of ICDAS score increases on the vestibular surfaces of teeth 15‐25 over the course of the study are shown in Table 1. At day 28, 3.2% of the teeth in the HAP group were already affected (ICDAS code 1: 3%, ICDAS code 2: 0.2%) compared to 3.6% of the fluoride control group (ICDAS code 1: 3.1%, ICDAS code 2: 0.5%). These figures steadily increased over time. At day 168, 19.6% of the teeth in the HAP group were affected (ICDAS code 1: 14.8%, ICDAS code 2: 4.8%) compared to 21% in the fluoride controls (ICDAS code 1: 14.2%, ICDAS code 2: 6.7%, ICDAS code 3: 0.1%).

**Table 1:** Number and severity of ICDAS score increases observed on teeth 15‐25 at day 28, day 84, and day 168 (PP dataset, N = 133)

Visit	Δ ICDAS	HAP test group		AmF/SnF_2_ control group		Total	
		Teeth (N)	%	Teeth (N)	%	Teeth (N)	%
Day 28	No increase	620	96.9	665	96.4	1285	96.6
	Δ ICDAS code 1	19	3.0	24	3.5	43	3.2
	Δ ICDAS code 2	1	0.2	1	0.1	2	0.2
	Total	640	100	690	100	1330	100
Day 84	No increase	573	89.5	611	88.6	1184	89.0
	Δ ICDAS code 1	58	9.1	59	8.6	117	8.8
	Δ ICDAS code 2	9	1.4	20	2.9	29	2.2
	Total	640	100	690	100	1330	100
Day 168	No increase	514	80.3	545	79.0	1059	79.6
	Δ ICDAS code 1	95	14.8	98	14.2	193	14.5
	Δ ICDAS code 2	31	4.8	46	6.7	77	5.8
	Δ ICDAS code 3	0	0	1	0.1	1	0.1
	Total	640	100	690	100	1330	100

Δ, difference; AmF, amine fluoride; HAP, hydroxyapatite; ICDAS, International Caries Detection and Assessment System; PP, per protocol; SnF, SnF_2_, see: https://en.wikipedia.org/wiki/Tin(II)_fluoride.

### 2.14 Effect of study site on the primary outcome measure

The effect of study site on the primary outcome measure Δ ICDAS score ≥1 at day 168 was evaluated by logistic regression analysis. It included the factor's study site, treatment group, and the interaction between the study site and treatment group. Due to small sample sizes, the data for the Dresden, Munich and Frankfurt study sites were pooled (N = 40 patients). The results revealed a significantly lower incidence of the primary outcome at day 168 (*P *<* *0.001) at the combined smaller centres (Dresden, Munich, and Frankfurt) when compared to the study centres in Regensburg (N = 72 patients) and Wuerzburg (N = 35 patients). However, there was no significant interaction between the study site and treatment group, proving that the factor study site did not significantly affect efficacy differences between the treatment groups (Tables 2 and 3).

**Table 2:** Occurrence of a caries increase ICDAS ≥code 1 compared to baseline (primary outcome) at day 168 at the different study centres (ITT dataset, N = 147)

Study center	Increase ICDAS code ≥1	HAP test group		AmF/SnF_2_ control group		Total	
		N	%	N	%	N	%
Regensburg	No	11	30.6	8	22.2	19	26.4
	Yes	25	69.4	28	77.8	53	73.6
Wuerzburg	No	4	23.5	3	16.7	7	20.0
	Yes	13	76.5	15	83.3	28	80.0
Dresden	No	11	78.6	11	91.7	22	84.6
	Yes	3	21.4	1	8.3	4	15.4
Munich	No	6	100	6	100	12	100
	Yes	0	0.0	0	0	0	0
Frankfurt	No	0	0.0	1	100	1	50.0
	Yes	1	100	0	0.0	1	50.0

AmF, amine fluoride; HAP, hydroxyapatite; ICDAS, International Caries Detection and Assessment System; ITT, intent to treat; SnF_2_, see: https://en.wikipedia.org/wiki/Tin(II)_fluoride.

**Table 3:** Increase in International Caries Detection and Assessment System score ≥1 at day 168 (logistic regression)

Parameter	Category	Estimate	*P*‐value
Treatment group	Control group	0.40	0.64
Combined centres	Combined centres	−2.66	0.0009
	Regensburg	−0.36	0.60
Age		−0.0708	0.52
Treatment Group of combined centres	Control group of combined centres	−1.10	0.38
	Regensburg control group	0.04	0.97

The result of a logistic regression analysis for the dichotomous primary study outcome Δ ICDAS score ≥1 at day 168, including the factors treatment group, center (study centres Frankfurt, Munich, and Dresden combined), age, and the interaction between the treatment group and center, is shown in Table 3. The analysis revealed only a significant effect for combined centres versus the reference category Wuerzburg. This indicates that the occurrence frequency of the primary study outcome at day 168 was significantly lower in the combined smaller centres (Dresden, Munich, Frankfurt) than in the larger study centres Wuerzburg and Regensburg. The interaction between the center and treatment was not significant (*P *=* *0.382) for the control group of combined centres (*P *=* *0.9686 for the Regensburg control group). Thus, it can be concluded that the efficacy of both treatments did not differ significantly between the study centres.

## 3 RESULTS

### 3.1 Patient recruitment and dropouts

Among a total of 281 screened individuals, 150 met the inclusion criteria and provided written informed consent and were recruited at the study centres in Wuerzburg (N = 36), Regensburg (N = 72), Dresden (N = 28), Munich (N = 12), and Frankfurt (N = 2).

**Figure 2:**
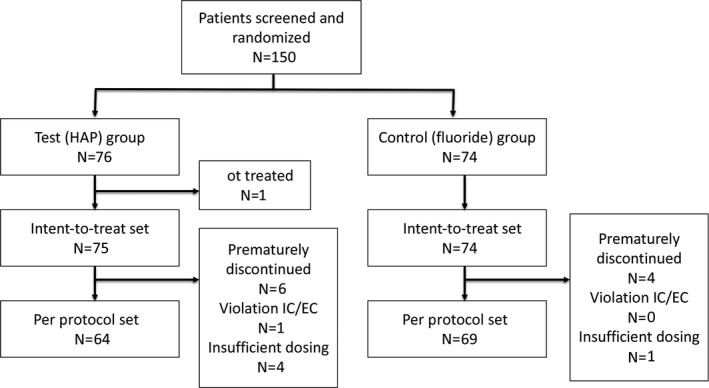
Consolidated standards of reporting trials (CONSORT) flow diagram. EC, ethics committee; HAP, hydroxyapatite; IC, informed consent

The first patient was included in the trial on 13 November 2013, and the last patient left the trial on 28 August 2016. Six patients in the test group and four patients in the control group terminated study participation prematurely due to lack of interest or not attending follow‐up appointments. One hundred and forty‐seven patients who received at least one dose of the assigned dentifrice, and who returned to at least the first re‐evaluation, were included in the ITT analysis. One hundred and thirty‐three study patients (64 test group/69 control group) finished the study per protocol (Figure 2). No significant problems or unintended effects related or unrelated to the use of the study dentifrices were reported.

### 3.2 Health status, age, and sex

All study patients were healthy. The mean age was 13.4 years ± 1.8 standard deviation (*SD*) for the HAP test group and 13.4 years ± 1.7 *SD* for the fluoride control group. The HAP test group consisted of 52.7% and the fluoride control group of 62.2% female patients.

### 3.3 Occurrence of International Caries Detection and Assessment System lesions ≥code 1 and ≥code 2

The occurrence of ICDAS lesions ≥code 1 (primary outcome) and ICDAS lesions ≥code 2 (secondary outcome) is depicted in Table 4. In the PP analysis, 54.7% of the HAP group patients and 60.9% of the fluoride control group patients showed the formation of at least one ICDAS lesion ≥code 1 during the 168‐day observation period. In the ITT analysis, the corresponding numbers were 56.8% for the patients in the HAP group and 61.9% for those in the fluoride control group. In the PP dataset the occurrence of at least one ICDAS lesion ≥code 2 was observed in 23.4% of the patients in the HAP group compared to 34.8% in the fluoride control group. In the ITT dataset, the corresponding numbers were 25.7% in the HAP group and 32.9% in the fluoride control group. Differences between the groups were not significant for both analysis sets.

**Table 4:** Occurrence of ICDAS lesions ≥code 1 and ≥code 2 within the 168‐day observation period (ITT and PP analysis)

Treatment group	ICDAS lesion code	PP analysis			ITT analysis		
		%	Patients with ICDAS lesions ≥code 1 and ≥code 2 (N)	Patients in the corresponding treatment group (N)	%	Patients with ICDAS lesions ≥code 1 and ≥code 2 (N)	Patients in the corresponding treatment group (N)
HAP test	≥1^a^	54.7	35	64	56.8	42	74
AmF/SnF_2_ control	≥1^a^	60.9	42	69	61.6	45	73
HAP test	≥2	23.4	15	64	25.7	19	74
AmF/SnF_2_ control	≥2	34.8	24	69	32.9	24	73

AmF, amine fluoride; HAP, hydroxyapatite; ICDAS, International Caries Detection and Assessment System; ITT, intent to treat; PP, per protocol; SnF, stannous fluoride.

a Primary outcome measure.

### 3.4 Non‐inferiority analysis

The difference between both experimental groups in terms of the percentage of study participants experiencing a new occurrence of at least one ICDAS lesion ≥code 1 (primary outcome) or at least one ICDAS lesion ≥code 2 (secondary outcome), including the corresponding one‐sided 95% confidence intervals, as displayed in Table 5. As the upper limits of the 95% confidence intervals for the primary outcome were well below the given non‐inferiority margin of Δ ≤ 20% for both analysis sets (PP: 8%, ITT: 9%), the HAP group was considered to be non‐inferior to the fluoride control.

**Table 5:** Difference between experimental groups regarding the occurrence of ICDAS lesions ≥code 1 and ≥code 2 within the 168‐day observation period (95% one‐sided confidence intervals)

Analysis	ICDAS lesion code	Proportion in risk difference	Exact lower one‐sided 95% confidence limit	Exact upper one‐sided 95% confidence limit^b^
PP analysis	≥1^a^	−0.062	−0.203	0.083
ITT analysis	≥1^a^	−0.048	−0.188	0.087
PP analysis	≥2	−0.114	−0.255	0.030
ITT analysis	≥2	−0.072	−0.202	0.068

ICDAS, International Caries Detection and Assessment System; ITT, intent to treat; PP, per protocol.

a Primary outcome measure.

b Upper one‐sided 95% confidence limit is markedly lower than the non‐inferiority margin of 0.20 (Δ = 20%), thus inferiority is rejected.

Regarding the secondary outcome (ICDAS lesion ≥code 2), the upper limits of the 95% confidence intervals were also substantially below the given non‐inferiority margin of 20% for both analysis sets (PP: 3%, ITT: 7%), again indicating that the HAP test group was non‐inferior to the fluoride control.

### 3.5 Plaque Index and Gingival Index

The results of the ITT analysis of the Pl and the GI data are shown in Table 6. The mean Pl and GI scores increased significantly (*P *<* *0.0001) from baseline to day 168 in both groups, but they were not significantly different between the groups at any time point.

**Table 6:** Plaque index and gingival index scores at baseline, day 28, day 84, and day 168 (ITT analysis)

Visit	HAP test group			AmF/SnF_2_ control group		
	N	Mean^a^	*SD*	N	Mean^a^	*SD*
Plaque index						
Baseline^b^	75	0.35	0.37	74	0.36	0.36
Day 28	74	0.65	0.58	72	0.76	0.56
Day 84	74	0.72	0.60	73	0.75	0.61
Day 168^b^	74	0.85	0.66	73	0.77	0.61
Gingival index						
Baseline^b^	75	0.29	0.36	74	0.37	0.41
Day 28	74	0.53	0.57	73	0.58	0.54
Day 84	74	0.51	0.53	73	0.66	0.55
Day 168^b^	74	0.70	0.56	73	0.77	0.59

AmF, amine fluoride; HAP, hydroxyapatite; ITT, intent to treat; *SD*, standard deviation; SnF_2_, see: https://en.wikipedia.org/wiki/Tin(II)_fluoride.

a Significant (*P *<* *0.0001) increase in the plaque index and gingival index over time from baseline to day 168 for both treatment groups (Friedman test).

b No significant differences between both treatment groups at baseline and day 168 (two‐sided Wilcoxon‐Mann‐Whitney test).

## 4 DISCUSSION

### 4.1 Methods

Caries detection and grading in this trial followed the principles of ICDAS‐II,^18^ an internationally‐established, state‐of‐the‐art caries assessment method that is particularly suitable and appropriate for the differentiation and grading of incipient enamel caries. Due to repeated examiner calibrations, the mean weighted kappa for interrater reliability increased from initially 0.75 for the first to 0.80 for the final calibration assessment, demonstrating an overall in the upper range of the kappa reliability scores reported by other controlled clinical trials and indicative of “substantial” agreement.^19^

### 4.2 Evaluation model and study population

Following the recommendations made by the International Consensus Workshop on Caries Clinical Trials in 2004,^23^ only high caries risk orthodontic patients were recruited. Despite regularly brushing with the assigned dentifrices, both experimental groups showed a considerable increase in enamel caries during the 168‐day observation period comparable in its magnitude to findings of other clinical trials.^13^, ^24^ In all comparisons made, and in particular regarding the development of more severe caries lesions ICDAS ≥code 2, the percentage of HAP group individuals affected by the new occurrence of an enamel caries lesion was consistently lower than the percentage of fluoride control group members (Table 4). However while proof of non‐inferiority could be established, the observed differences failed to reach significance. Due to the lack of a negative control group in the present trial for ethical reasons, it is difficult to determine the true extent of caries inhibition provided by the evaluated dentifrices to the study patients.

In a more recent caries trial by Sonesson et al. assessing a comparable cohort of 424 adolescent orthodontic patients, the regular use of a standard low‐dose 1450 ppm fluoride dentifrice resulted in a significantly higher incidence of white spot enamel lesions (26.6% vs 18.1%) when compared to the use of a highly‐concentrated 5000 ppm fluoride dentifrice.^25^ While this result indicates that a low‐dose fluoride dentifrice might not provide optimal protection for caries‐active orthodontic patients, we cannot conclude that it did not confer any measurable caries‐inhibiting effect, as 141 of overall 192 patients in the low‐dose fluoride group evaluated by Sonesson et al. did not develop any new white spot lesions during the observation period. In the present study, approximately 40% of the study participants in both groups were not affected by the new occurrence of a caries lesion. This suggests that while all of them shared the common risk factor of elevated salivary levels of caries‐promoting *mutans streptococci*, the individual strength of the cariogenic challenge differed considerably. This might have been related to possible individual differences regarding salivary flow, buffer capacity, and other caries‐modulating factors not controlled by the study design. Nevertheless, for some study participants, at certain sites the magnitude of the acidic challenge exceeded the limits of the caries‐protective properties of microcrystalline HAP and low‐dose fluoride, which might have masked possible differences in the caries‐preventive efficacy of the evaluated dentifrices under less acidic conditions.

### 4.3 Data analysis

Whether the occurrence frequency of ICDAS code 1 enamel caries lesions used in the present study is the most suitable primary endpoint for a non‐inferiority caries trial is subject to discussion. However, the adjunctive analysis of the PP dataset regarding the frequency and severity of the occurrence of enamel caries lesions during the observation period, as depicted in Table 1, only endorsed the identified absence of relevant differences between the groups.

The data for the secondary outcomes (PI and GI) further confirmed the findings of preceding studies, reporting a significant increase in gingival inflammation and bacterial plaque mass after the onset of orthodontic therapy with fixed appliances.^13^, ^24^ Differences between the groups regarding PI and GI were not significant for any of the evaluated time points, which was also in good agreement with the results of a previous trial comparing the plaque‐ and gingivitis‐reducing properties of a fluoride‐free HAP test dentifrice and a fluoridated amine fluoride (AmF)/SnF_2_control in a study cohort of patients suffering from mild to moderate periodontitis.^10^

### 4.4 Outlook

While the safety of fluoride‐based caries prevention has been firmly established by numerous studies,^16^ dosage and toxicity aspects must always be considered. A caries‐inhibiting increase in the applied fluoride dosage in caries‐risk patients, as described by Sonesson et al.,^25^ might thus not be feasible in infants and children up to the age of 8 years due to the associated risk for the development of dental fluorosis. Although not verified by clinical studies thus far, an increase in the dosing or application frequency of HAP toothpaste might also potentially boost the caries‐inhibition efficacy in caries‐active patients, as HAP is a potent buffer under acidic conditions that is able to neutralize organic acids. Unlike fluorides, a HAP dosage increase is not affected by any toxicity issues, even in infants and children, as HAP is the major mineral phase of all human hard tissues.^5^

### 4.5 Conclusions

The data of this 6‐month, clinical non‐inferiority trial demonstrate, that in highly caries‐active orthodontic patients, the impact of the regular use of a fluoride‐free, microcrystalline HAP dentifrice on caries progression is not significantly different from the use of a fluoridated (350 ppm of AmF/1050 ppm of SnF_2_) toothpaste. An evidence‐based judgement regarding the general suitability of microcrystalline HAP as a substitute or adjunct to fluorides in clinical caries prevention might only be possible after the availability of further data derived from clinical trials in study cohorts of diverse age and varying magnitude of the cariogenic challenge.
